# Persistent Lactatemia in Mauriac Syndrome

**DOI:** 10.1155/2024/5599984

**Published:** 2024-05-07

**Authors:** Nada El Tobgy, Laura Hinz

**Affiliations:** University of Calgary, Calgary, Alberta, Canada

## Abstract

Mauriac syndrome is a rare disorder that occurs in patients with type 1 diabetes mellitus (T1DM) with glucose levels significantly above target, characterized by hepatomegaly, growth delay, and cushingoid features. Another distinguishing feature of Mauriac syndrome is persistent lactatemia during diabetic ketoacidosis (DKA) management. We present a case of an 18-year-old patient with T1DM who presented in DKA and then developed elevated lactate levels leading to a diagnosis of Mauriac syndrome. The cause of the persistent lactatemia is not well understood though it is likely related to glycogenic hepatopathy causing hepatomegaly, abnormalities in glucose metabolism, and subsequent inappropriate lactate production. Since the liver changes seen in Mauriac syndrome are reversible with optimal blood glucose control, these patients should be connected to intensive psychosocial and medical support to help them improve their blood glucose levels.

## 1. Introduction

Mauriac syndrome is a rare disorder that occurs in patients with type 1 diabetes mellitus (T1DM) with glucose levels significantly above target particularly in adolescents and young adults, though it can occur at any age [1]. It can occur equally in males and females. It is associated with brittle glycemic control, high total daily doses of insulin, and recurrent episodes of diabetic ketoacidosis (DKA). It has become less common with the advent of accessible and affordable intensive insulin therapy using premixed basal and bolus insulins as well as continuous subcutaneous insulin infusions [2]. The main hallmarks of this syndrome are hepatomegaly with altered liver enzymes, growth delay, delayed puberty, and cushingoid features [3]. The altered liver enzymes are due to hepatic glycogenosis caused by chronically elevated blood glycose levels [4, 5]. The liver dysfunction can also lead to persistent lactatemia that can transiently worsen with DKA and persist after resolution of DKA [5–7].

We present a case of an 18-year-old patient with T1DM admitted with DKA who was found to have persistently elevated lactate and hepatomegaly, then was subsequently diagnosed with Mauriac syndrome based on their clinical and biochemical findings.

## 2. Case Presentation

An 18-year-old nonbinary (sex-assigned at birth was female) patient with T1DM with an A1c of 10.9% presented to our tertiary care center with DKA.

On history, they were diagnosed with T1DM at the age of 9 and had multiple prior presentations with DKA earlier that year. They used Degludec 30 units in the morning and Aspart insulin with meals with an insulin to carbohydrate ratio of 1 : 5. They had been previously on a pump, which was discontinued since the patient was unable to consistently test their capillary glucose levels, and so were no longer eligible for provincial funding to cover the cost of the pump. During an admission a year prior with DKA, this patient had also been noted to have hepatomegaly of unclear etiology on prior CT imaging of their abdomen, despite extensive workup. This included infectious and autoimmune investigations such as hepatitis B and C screening, HIV and CMV testing, negative ANA, rheumatoid factor, smooth muscle antibody, anti-liver kidney microsome, anti-TTG, negative alpha-1 antitrypsin testing, and a negative ceruloplasmin. In addition, the patient struggled with multiple mental health and developmental concerns, including anxiety/depression, suicidal ideation, borderline personality disorder traits, and possible autism spectrum disorder, and had two prior hospitalizations under Psychiatry for suicidality.

Preceding their admission, the patient had a viral gastrointestinal illness and was unable to manage their own insulin administration at home. On admission, they presented with an elevated anion gap of 35 mmol/L and hyperglycemia with serum blood glucose of 41.6 mmol/L, as well as an elevated lactate of 5.8 mmol/L that increased to 7.8 mmol/L on repeat blood work. Their DKA was treated with intravenous insulin infusion and hydration. As their anion gap closed, their lactate initially improved to 4.2 mmol/L but then quickly increased to a peak of 8.4 mmol/L. They were assessed for type A lactic acidosis (hypoperfusion and tissue damage) but no cause was found. Their lactate spontaneously decreased and reached a nadir of 2.4 mmol/L but never normalized. At this point, the inpatient Endocrinology service was consulted to assist with the etiology of the elevated serum lactate in this patient.

On exam, the patient was found to have cushingoid features, including preauricular and central adiposity. However, they did not have hypertension or violaceous striae. Their height was at the 11th percentile for their age with a BMI of 25.3 kg/m^2^. During the assessment for type A lactic acidosis, an abdominal CT scan with enhancement was done to rule out ischemic bowel and found hepatomegaly suspicious for congenital hepatopathy based on the radiologist's interpretation.

Further investigations showed an A1c of 10.9% and elevated liver enzymes including an AST 99 U/L, ALT 81 U/L, and ALP 140 U/L. They also had elevated triglycerides of 4.65 mmol/L. They had a normal TSH and fT4. Due to the presence of cushingoid features, a 1 mg dexamethasone overnight suppression test was done after resolution of their DKA and revealed a nonsuppressed cortisol of 314 nmol/L.

Following treatment for DKA, the patient's symptoms resolved, and they were discharged with plans for close follow-up with their outpatient Endocrinologist and Diabetes Educator for reconsideration for insulin pump therapy. Their lactate reached a nadir of 2.4 mmol/L but never normalized.

After discharge, the patient struggled with checking their blood glucose regularly and as a result was not a candidate for an insulin pump. Although they initially denied hypoglycemic episodes, collateral history from their grandmother revealed that the patient would need to be fed juice and dextrose tablets to treat episodes of hypoglycemia up to 3-4 times per week. The patient is currently working with diabetes educator on improving their blood glucose management, though continues to struggle with follow-up. Lactate levels and repeated imaging of the patient's liver have not yet been repeated, though the patient has been given requisitions for further testing and imaging.

## 3. Discussion

Mauriac syndrome was first described in 1930 in children and adolescents with T1DM who presented with hepatomegaly and growth/developmental delay [8]. It has become much less common with the advent of modern preparations of long and short acting insulins [1, 3]. The exact cause of Mauriac syndrome is unknown, though it is hypothesized to be caused by poor uptake and utilization of glucose by tissues, decreased insulin-like growth factor-1 and growth hormone levels, and hypercortisolism, that then result in developmental delays [3, 4, 6, 9]. As this is a very rare disorder, there are no formal diagnostic criteria for Mauriac syndrome.

There are two phenotypes of Mauriac syndrome described in the literature [3]. The first subtype is associated with periods of hypo- and hyperglycemia and cushingoid features as a result of inconsistent insulin dosing. The second subtype is associated with persistent hyperglycemia due to underdosing insulin without cushingoid features [3]. As this patient presented with T1DM with elevated Hemoglobin A1c, hepatomegaly, and cushingoid features, they were diagnosed with Mauriac syndrome, falling into the first subtype. Of note, the 1 mg dexamethasone overnight suppression test was done in the inpatient setting and thus is likely to confounded by acute illness. Repeat testing is planned in the outpatient setting but the patient has not been able to complete it.

One of the characteristic features of Mauriac syndrome is glycogenic hepatopathy, marked by hepatomegaly and abnormal liver enzymes. Although its pathophysiology is not completely understood, it is thought to be caused by elevated blood glucose levels and administration of supraphysiologic insulin doses to control hyperglycemia [9, 10]. The high insulin and glucose levels both stimulate glycogenesis in the liver, resulting in excessive glycogen storage and hepatomegaly. Glycogen production can persist even after the decline of insulin levels [10]. It is unclear why only some patients with chronically elevated blood glucose levels develop glycogenic hepatopathy. Several gene mutations in enzymes involved in glycogen metabolism, such as PHGK2 [11], have been investigated as culprits, but none thus far show clear association with the disease [4, 9, 10, 12].

The gold standard method of diagnosing glycogenic hepatopathy is on histology through a liver biopsy, which is especially useful in distinguishing it from metabolic dysfunction-associated steatotic liver disease (MASLD) [2]. Unlike MASLD, patients with glycogenic hepatopathy do not exhibit progressive liver fibrosis. Furthermore, glycogenic hepatopathy associated with Mauriac syndrome is reversible with adequate BG control without any liver fibrosis or scarring [2, 12–14].

One feature of Mauriac syndrome that has been noted in the literature is the presence of persistently elevated serum lactate levels. Type A lactic acidosis is caused by hypoperfusion of peripheral tissue and end-organ dysfunction, while type B is characterized by hepatic dysfunction in the synthesis or clearance of lactate. The cause of persistent lactatemia in Mauriac syndrome is not currently well understood. One current hypothesis suggests that chronic hepatic glycogen overload reduces gluconeogenesis during periods of excess insulin availability during DKA treatment, thus shunting glucose to lactate-producing pathways [15]. Other hypotheses postulate the presence of a genetic defect in glycogen metabolism that predisposes an individual to glycogenic hepatopathy and subsequent sequelae from the disease [5, 16].

Although liver changes associated with Mauriac syndrome are reversible with adequate BG control, the neurodevelopmental delay associated with the syndrome makes this challenging. Furthermore, patients with Mauriac syndrome often come from backgrounds lacking adequate psychosocial support and health literacy. As demonstrated by this case, an increased effort to connect patients with Mauriac syndrome to holistic supports, including social work, psychology/psychiatry, and community resources, is paramount to preventing further episodes of DKA and reversing liver changes.

## 4. Conclusion

This case demonstrates how persistent lactatemia in the setting of DKA in a patient with T1DM can alert clinicians to consider Mauriac syndrome as a diagnosis. Early identification of these patients is beneficial in connecting them with more intensive psychosocial support to achieve in-target BG levels and resolution of their glycogenic hepatopathy.

## Data Availability

This is a case report describing one patient. The patient has signed a release form to have their data published anonymously for this case report. The data were taken from the patient's electronic medical record through Alberta Health Services. The data supporting the conclusions are in the body of this manuscript. If further data are required, then please contact the corresponding author at Nada.ElTobgy@albertahealthservices.ca for anonymized data.
